# Cefepime-zidebactam therapy for extensively drug-resistant *Pseudomonas aeruginosa* and *Klebsiella pneumoniae* infection as a bridge to liver transplantation

**DOI:** 10.1093/jacamr/dlaf129

**Published:** 2025-07-17

**Authors:** Shemual Tsai, Masayuki Nigo, Donghoon Kang, Rodrigo P Baptista, Pranita D Tamma, Emily Jacobs, Yehudit Bergman, David W Victor, Ashton A Connor, Ashish Saharia, R Mark Ghobrial, Cesar A Arias, William R Miller

**Affiliations:** Department of Pharmacy, Houston Methodist Hospital, Houston, TX, USA; Division of Infectious Diseases and Center for Infectious Diseases, Houston Methodist Hospital and Houston Methodist Research Institute, Houston, TX, USA; Department of Medicine, Weill-Cornell Medical College, New York, NY, USA; Division of Infectious Diseases and Center for Infectious Diseases, Houston Methodist Hospital and Houston Methodist Research Institute, Houston, TX, USA; Division of Infectious Diseases and Center for Infectious Diseases, Houston Methodist Hospital and Houston Methodist Research Institute, Houston, TX, USA; Department of Medicine, Weill-Cornell Medical College, New York, NY, USA; Department of Pediatrics, Johns Hopkins University School of Medicine, Baltimore, MD, USA; Department of Pediatrics, Johns Hopkins University School of Medicine, Baltimore, MD, USA; Department of Pediatrics, Johns Hopkins University School of Medicine, Baltimore, MD, USA; Department of Medicine, Weill-Cornell Medical College, New York, NY, USA; Sherrie and Alan Conover Center for Liver Disease and Transplantation, JC Walter Jr Transplant Center, Houston Methodist Hospital, Houston, TX, USA; Sherrie and Alan Conover Center for Liver Disease and Transplantation, JC Walter Jr Transplant Center, Houston Methodist Hospital, Houston, TX, USA; Department of Surgery, Houston Methodist Hospital, Houston, TX, USA; Sherrie and Alan Conover Center for Liver Disease and Transplantation, JC Walter Jr Transplant Center, Houston Methodist Hospital, Houston, TX, USA; Department of Surgery, Houston Methodist Hospital, Houston, TX, USA; Sherrie and Alan Conover Center for Liver Disease and Transplantation, JC Walter Jr Transplant Center, Houston Methodist Hospital, Houston, TX, USA; Department of Surgery, Houston Methodist Hospital, Houston, TX, USA; Department of Surgery, Weill Cornell Medical College, New York, NY, USA; Division of Infectious Diseases and Center for Infectious Diseases, Houston Methodist Hospital and Houston Methodist Research Institute, Houston, TX, USA; Department of Medicine, Weill-Cornell Medical College, New York, NY, USA; Division of Infectious Diseases and Center for Infectious Diseases, Houston Methodist Hospital and Houston Methodist Research Institute, Houston, TX, USA; Department of Medicine, Weill-Cornell Medical College, New York, NY, USA

## Abstract

**Background:**

Infections due to antimicrobial-resistant Gram-negative organisms present increasingly difficult therapeutic challenges, especially in the presence of metallo-β-lactamases. We present the case of a patient with cholangitis due to *Pseudomonas aeruginosa* and *Klebsiella pneumoniae* isolates that developed cefiderocol resistance on therapy treated successfully with cefepime-zidebactam.

**Methods:**

Serial clinical isolates recovered from biliary fluid and ascitic fluid were tested for susceptibility to cefiderocol, aztreonam-avibactam, cefepime-taniborbactam, cefepime-zidebactam, and cefiderocol-xeruborbactam by broth microdilution. Whole-genome sequencing was performed to identify resistance determinants. An emergency investigational new drug application was authorized by the United States Food and Drug Administration for the compassionate use of cefepime-zidebactam based on susceptibility test results.

**Results:**

Index isolates of *P. aeruginosa* (IMP positive) and *K. pneumoniae* (NDM-5, OXA-232 positive) tested susceptible to cefiderocol by disk diffusion in the clinical microbiology laboratory. The patient was treated with a regimen of cefiderocol and eravacycline, with persistent fever and development of hepatic microabscesses on imaging. Compassionate use cefepime-zidebactam therapy was initiated the day prior to liver transplantation and continued for a total of 14 days due to positive ascitic fluid cultures obtained during the operation. The *K. pneumoniae* and *P. aeruginosa* were cefiderocol resistant by broth microdilution. Cefepime-zidebactam remained active with MICs of 8/8 mg/L and 32/32 mg/L, respectively. The patient did well post-transplant and resumed chemotherapy.

**Conclusion:**

Antimicrobial therapy with cefepime-zidebactam along with source control allowed successful liver transplantation in a patient with cefiderocol-resistant *K. pneumoniae* and *P. aeruginosa*. Cefepime-zidebactam may be a therapeutic option for extensively drug-resistant Gram-negative organisms.

## Introduction

*Pseudomonas aeruginosa* and *Klebsiella pneumoniae* are important nosocomial pathogens that may display resistance to multiple antibiotics due to both intrinsic and acquired resistance determinants. Antibiotic therapy options are further limited in metallo-β-lactamase-producing (MBL) organisms, which display resistance to the current United States FDA approved β-lactam/β-lactamase inhibitors (BL/BLI) ceftolozane/tazobactam, ceftazidime/avibactam, meropenem/vaborbactam, and imipenem-cilastatin-relebactam.^1^ While cefiderocol generally demonstrates *in vitro* activity against these isolates, both *P. aeruginosa* and *K. pneumoniae* can rapidly develop cefiderocol resistance via TonB-dependent siderophore receptor mutations.^2–6^

Here, we present the case of a patient that underwent chemotherapy and liver transplantation in the setting of intra-abdominal infection with a *P. aeruginosa* producing imipenemase (IMP) enzymes and a *K. pneumoniae* isolate producing New Delhi metallo-β-lactamases (NDM) and OXA-48-like carbapenemases. Both organisms developed cefiderocol non-susceptibility during cefiderocol treatment. The patient was ultimately cured of infection with the use of cefepime-zidebactam and liver transplantation.

## Methods

Cefepime-zidebactam was obtained from Wockhardt under an emergency Investigational New Drug application (eIND) authorized by the FDA. The protocol was approved by the Institutional Review Board at Houston Methodist (PRO00038383), and patient consent was obtained. Clinical isolates of *P. aeruginosa* and *K. pneumoniae* were recovered from the clinical microbiology laboratory at Houston Methodist Hospital for susceptibility testing as a part of the protocol. Isolates were grown on MacConkey agar (*K. pneumoniae*) or Cetrimide agar (*P. aeruginosa*) and stored in Brucella broth plus 15% glycerol at −80°C. Antimicrobial susceptibility testing was performed in triplicate via broth microdilution at Johns Hopkins University School of Medicine using frozen antimicrobial panels in accordance with Clinical and Laboratory Standards Institute (CLSI) guidelines with interpretive criteria based on the M100 35th edition published in 2025.

Genomic DNA was isolated from the recovered isolates and sequenced on a MiSeq platform (Illumina, Inc.) for short reads and MinION (Oxford Nanopore) for long reads to perform hybrid assembly and close genomes for plasmid identification. Genomic data were analysed using a custom pipeline. The whole genome sequencing data are available at the National Center for Biotechnology Information (NCBI) website, Bioproject accession number PRJNA1225750.

## Results

**Figure 1.**
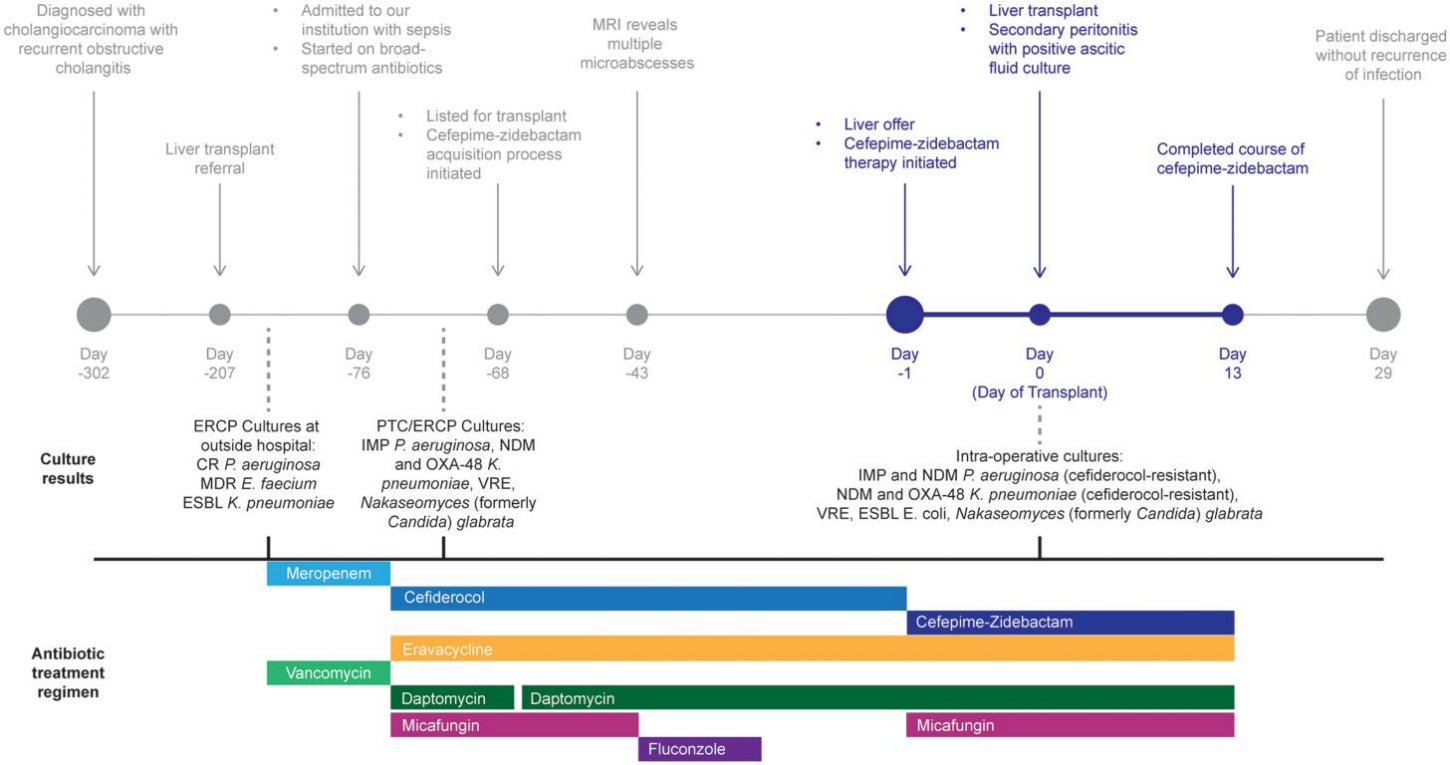
Clinical timeline, culture results, and antimicrobial therapy. Day of transplant is given as day zero. Antimicrobials are shaded by class/spectrum: blue, β-lactams and β-lactam/β-lactamase inhibitors; orange, tetracyclines; green, antibiotics with Gram-positive activity only; purple, antifungals. CR, carbapenem-resistant; ESBL, extended spectrum β-lactamse; ERCP, endoscopic retrograde cholangiopancreatography; IMP, active-on-imipenem; MDR, multidrug-resistant; NDM, New Delhi Metallo-β-lactamase; OXA, oxacillinase; PTC, percutaneous transhepatic cholangiography.

A woman in her 70 s who recently immigrated from Pakistan was diagnosed with cholangiocarcinoma resulting in recurrent obstructive cholangitis. Her initial care took place at an outside hospital, where she received chemotherapy with gemcitabine, cisplatin, and durvalumab. She developed cholangitis, was started on meropenem and vancomycin, and her biliary fluid cultures obtained during endoscopic retrograde cholangiopancreatography (ERCP) grew a carbapenem-resistant *P. aeruginosa*, vancomycin-resistant *Enterococcus faecium* resistant to linezolid and with a daptomycin MIC of 2 mg/L, and carbapenem-resistant *K. pneumoniae*. She presented as a transfer to our institution where cefiderocol 2 g IV every 8 h, eravacycline 1 mg/kg IV every 12 h, daptomycin 10 mg/kg IV every 24 h, and micafungin 100 mg IV every 24 h was administered. Percutaneous transhepatic cholangiogram with drain placement occurred 5 days after admission, followed by another ERCP with stent exchange the following day. New biliary cultures again grew IMP-producing *P. aeruginosa* and NDM and OXA-48-like producing *Klebsiella pneumoniae*. The patient also grew additional organisms beyond the Gram-negative infection discussed; specific details are included in Figure 1.

Despite antimicrobial therapy, she continued to have intermittent episodes of fever and rigours. Four weeks into treatment, MRI of the abdomen identified several new hepatic microabscesses up to 14 millimetres in size. After discussion between transplant infectious diseases, the surgical transplant team, and oncology, liver transplantation was considered as the only potentially curative treatment option for her cholangiocarcinoma and uncontrolled infection.

With concerns for the emergence of cefiderocol resistance due to persistent fevers and development of microabscesses while on antimicrobial therapy, we initiated the process to obtain compassionate use cefepime-zidebactam. This antibiotic combines the cephalosporin cefepime with a bicyclo-acyl hydrazide β-lactamase inhibitor zidebactam. Importantly, zidebactam also possesses activity against PBP2 from many Gram-negative organisms, with multiple PBP inhibition resulting in an enhancer effect that leads to synergistic killing.^7,8^ Cefepime-zidebactam susceptibility testing was performed for both the *P. aeruginosa* isolate (MIC of 32/32 mg/L, susceptible) and *K. pneumoniae* isolate (MIC of 8/8 mg/L, susceptible, Table 1), both considered susceptible based on the investigational susceptible-only breakpoint of ≤64/64 mcg/mL established by CLSI. The patient received an organ offer for liver transplantation, and the decision was made to change from cefiderocol to cefepime-zidebactam 2 g IV every 8 h the evening prior to transplant to optimize antimicrobial exposure at the time of surgery as she was continuing to have fevers. Daptomycin and eravacycline were continued.

**Table 1.** Antimicrobial susceptibility testing results via broth microdilution of *Klebsiella pneumoniae* and *Pseudomonas aeruginosa* isolates before and during cefepime-zidebactam therapy

Isolate	MIC (mg/L) and susceptibility interpretation							
	Before cefepime-zidebactam therapy		During cefepime-zidebactam therapy		Before cefepime-zidebactam therapy		During cefepime-zidebactam therapy	
Species	*Klebsiella pneumoniae*		*Klebsiella pneumoniae*		*Pseudomonas aeruginosa*		*Pseudomonas aeruginosa*	
Aztreonam/avibactam^a^	8/4	S	8/4	S	—	—	—	—
Cefiderocol^b^	128	R	128	R	>128	R	128	R
Cefepime/taniborbactam^c^	16/4	S	16/4	S	>64/4	R	>64/4	R
Cefepime/zidebactam^d^	8/8	S	8/8	S	32/32	S	16/16	S
Cefiderocol/xerubobactam^e^	0.25/8	S	0.25/8	S	64/8	R	64/8	R
Ceftolozane/tazobactam^f^	—	—	—	—	>128/4	R	>128/4	R
Ceftazidime/avibactam^g^	>128/4	R	>128/4	R	>128/4	R	>128/4	R
Imipenem/relebactam^h^	64/4	R	64/4	R	64/4	R	64/4	R
Meropenem/vaborbactam^i^	>128/8	R	>128/8	R	—	—	—	—

R, resistant; S, susceptible.

^a^Based on the European Committee on Antimicrobial Susceptibility Testing aztreonam/avibactam susceptibility breakpoint of ≤8/4 mg/L; a CLSI aztreonam/avibactam susceptibility breakpoint is not available.

^b^Based on the CLSI cefiderocol susceptibility breakpoint of ≤4 mg/L for both Enterobacterales and *P. aeruginosa*.

^c^Susceptibility based on a cefepime/taniborbactam investigational breakpoint of ≤16/4 mg/L.

^d^Susceptibility based on a CLSI-endorsed cefepime/zidebactam investigational breakpoint of ≤64/64 mg/L.

^e^Susceptibility based on the CLSI cefiderocol susceptibility breakpoint of ≤4 mg/L (without xeruborbactam); susceptibility breakpoints for cefiderocol/xeruborbactam not available.

^f^Susceptibility breakpoints based on CLSI breakpoint of ≤4/4 for *P. aeruginosa*.

^g^Susceptibility breakpoints based on CLSI breakpoint of ≤8/4 for both Enterobacterales and *P. aeruginosa*.

^h^Susceptibility breakpoints based on CLSI breakpoint of ≤1/4 mg/L for Enterobacterales and ≤2/4 mg/L for *P. aeruginosa*.

^i^Susceptibility breakpoints based on CLSI breakpoint of ≤4/8 mg/L for Enterobacterales.

The patient underwent complete hepatectomy with orthotopic liver transplantation. The Model for End-Stage Liver Disease score was 29 at time of transplant. Cultures taken from the biliary fluid and bile duct stent grew IMP-producing *P. aeruginosa* resistant to all newer BL/BLIs and cefiderocol, intermediate to colistin, and resistant to all other antibiotics including aminoglycosides and multi-drug resistant *K. pneumoniae* susceptible to eravacycline (MIC 0.5 mg/L, Table 1). Although source control for the liver abscess was achieved with removal of the native liver, the patient had secondary peritonitis with intraoperative ascitic fluid cultures growing *P. aeruginosa* and *K. pneumoniae*. Whole-genome sequencing of the new peritoneal isolates showed the *P. aeruginosa* belonged to ST4936 and possessed *bla*_PDC-35_ (encoding the intrinsic Pseudomonal cephalosporinase), along with the acquired β-lactamase genes *bla*_IMP-1_, *bla*_OXA-10_, and *bla*_PAC-1_. The *K. pneumoniae* belonged to ST437 (a member of the high-risk clonal group ST258), and carried the *bla*_SHV-11_, *bla*_CTX-M-15_, and *bla*_NDM-5_ genes. The decision was made to continue cefepime-zidebactam as eravacycline would not have activity against the *P. aeruginosa* isolate and polymyxins have shown limited efficacy as monotherapy with significant toxicities.

While continuing on cefepime-zidebactam, her clinical status improved and she was transferred from the intensive care unit to a regular floor eleven days after her transplant. The patient completed 14 days of cefepime-zidebactam for peritonitis. Computed tomography of the abdomen two weeks after transplant was notable for a small amount of perihepatic/subdiaphragmatic fluid without evidence of infection. The patient was off antibiotic therapy for approximately 6 weeks without recurrence of infection when she resumed chemotherapy for underlying cholangiocarcinoma.

## Discussion

Antibiotic resistance is a public health emergency that threatens the ability to perform lifesaving treatments such as organ transplantation. Particularly concerning is the increasing frequency of infections due to Gram-negative organisms that produce MBLs.^1^ In the present case, the infecting *P. aeruginosa* isolate was resistant to all currently available antibiotics, and additionally resistant to several BL/BLIs currently under development including cefepime-taniborbactam and cefiderocol-xeruborbactam. In conjunction with surgical source control, effective antimicrobial therapy with cefepime-zidebactam allowed for successful liver transplantation.

Currently, there are four case reports describing successful use of cefepime-zidebactam as compassionate treatment, all of which were carbapenem-resistant *P. aeruginosa* from India.^9–12^ Three of the four isolates produced NDM (in the other isolate no carbapenemase testing was described), and all patients failed polymyxin B-based therapy prior to the initiation of cefepime-zidebactam (MIC range 8/8–16/16 mg/L). The current case highlights several important aspects when considering treatment options for extensively drug-resistant pathogens. First, cefepime-zidebactam was successful as salvage therapy in the setting of treatment emergent resistance on cefiderocol. While removal of the infected native liver was crucial for source control, ascitic fluid sampled at the time of surgery grew both *P. aeruginosa* and *K. pneumoniae*. Thus, adjunctive antimicrobial therapy was critical for ensuring a successful transplant outcome. While eravacycline retained activity against the *K. pneumoniae* isolate and may have contributed to the successful outcome, it would not be active against the *P. aeruginosa* isolate. Second, zidebactam, due to the PBP2 activity, was able to potentiate cefepime when inhibitor-only agents such as taniborbactam (due to the presence of IMP) and xeruborbactam were unable to restore activity for their partner β-lactam. Clinical success was also seen when using cefepime-zidebactam against an isolate with an MIC of 32/32 mg/L, and it is critical to understand treatment outcomes for patients whose isolates are near the susceptible-only breakpoint of 64/64 mg/L. Thus, cefepime-zidebactam may be a potential option for cefiderocol-resistant *P. aeruginosa* infections and against taniborbactam ‘escape variants’ such as IMP-producers where there may be limited treatment options.^13^

While biliary penetration of cefepime-zidebactam has not been specifically studied, data on the individual components may suggest favourable penetration. Cefepime, as a 2 gram dose, has mean concentrations of 15.5 and 19.8 mcg/mL in bile, exceeding the MIC susceptibility breakpoint of most Gram-negative pathogens.^14^ Although zidebactam has not been formally studied with bile pharmacokinetics, it shares physiochemical properties of drugs that theoretically have higher biliary concentrations. Zidebactam is hydrophilic (predicted logP approximately −3), polar (polar surface area of 157 Å^2^), and has a molecular weight >300 g/mol—all of which are characteristics described of drugs with good biliary penetration.^14,15^ With this in mind, cefepime-zidebactam may be an antimicrobial option for biliary-related infections, similar to our patient case report.

In summary, we report the successful use of cefepime-zidebactam in the treatment of MBL-producing *P. aeruginosa* and *K. pneumoniae* in a patient undergoing liver transplantation. Although further clinical outcomes data are needed, this combination presents a potential treatment option for Gram-negative organisms with an extensive drug resistance phenotype.

## Data Availability

The whole genome sequencing data are available at the National Center for Biotechnology Information (NCBI) website, Bioproject accession number PRJNA1225750.
